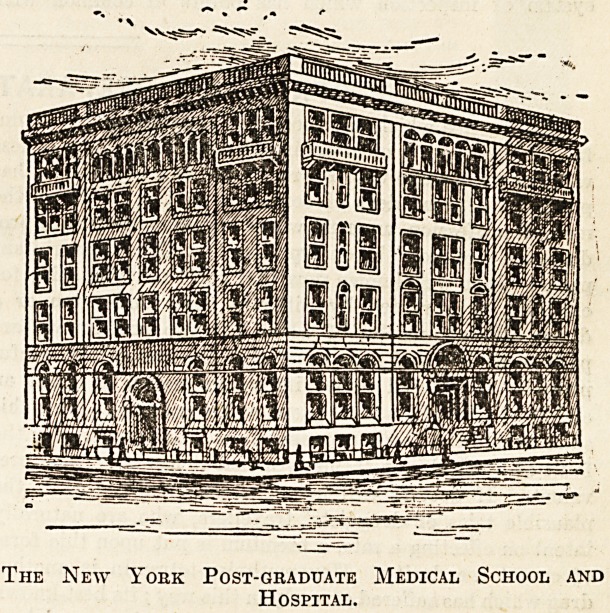# Graduate Study in America

**Published:** 1909-04-24

**Authors:** 


### HOSPITAL ADMINISTRATION.

### CONSTRUCTION AND ECONOMICS.

### GRADUATE STUDY IN AMERICA.

#### THE NEW YORK POST-GRADUATE MEDICAL SCHOOL AND HOSPITAL.

In a former article we briefly outlined the work and development of the excellent graduate study hospitals and schools which are to be found at St. Petersburg and Moscow, and pointed out that to the latter institutions must belong the credit of having been the pioneers in a movement the importance of which is only gradually becoming realised in England. The Russian schools are, however, closely rivalled, in point of priority, by the large and, of its kind, certainly the best equipped post-graduate medical school and hospital, Second Avenue and Twentieth Street, New York. We have lately had the pleasure of going over this fine institution, and an account of its history, progress, and work is an inevitable introduction to a consideration of the advance of the "graduate study ideal" in America.

Unlike the Moscow and St. Petersburg institutions, the New York school started in a small way, entirely unassisted by State grants or the support of great notabilities. It made up for these deficiencies by possessing from its inception the invaluable services of that keen enthusiast, Dr. Roosa, whose name easily heads the list of those who have taken up the cause of the graduate student. It was he who coined the compound "Post-Graduate," which, notwithstanding its suspicion of tautology, has succeeded in gaining a measure of respectful recognition; it was he who in 1882 founded the New York School and Hospital, and for more than twenty-four years continued its staunch supporter and friend. As early as 1875, a year after the Russian project was mooted, but two years before the St. Petersburg school was actually started, the Council of the University of the City of New York created a post-graduate faculty in its medical school; but this beginning was inauspicious. The classes could not be considered proper graduate classes, as they were attended by undergraduates as well as by matriculates.* Dr. Roosa, who justly appreciated the desirability of strictly limiting such classes to graduates, set himself strenuously to supply the want that existed. With the help of some of his colleagues who shared his views, and who, like him, had resigned from the University Faculty to be free to devote themselves whole-heartedly to the development of the graduate study ideal, he commenced work in the Chickering Hall on Fifth Avenue. His classes and clinics proved so popular that it was soon found necessary to organise the school on a definite basis, and after a time the present hospital and school were started. The inception of the school and hospital was the work of private enterprise; both have been supported during the quarter of a century which has elapsed since their foundation by private donations, the unselfishness of the staff of professional workers, and the charity of lay friends. Last year the school lost the help and support of its able founder; but before his death Dr. Roosa was able to see the realisation of a part, at least, of his ideal in the solid success and admirable progress of the hospital.

#### The Present Institution.

The institution on Second Avenue and Twentieth Street is a large, seven-storied block, plain but impressive in its solidity. In this building, under one roof, are housed both the school and the hospital—an excellent arrangement which has everything to recommend it. On the ground floor are the school quarters; the administrative offices; a large, airy, and well stocked reading-room; a medium sized demonstration theatre; and side rooms. On the upper floors are located the larger theatres, including a very large and excellently lighted operating theatre, the eye and ear operating room, a fine laboratory for chemico-pathological purposes, with attached pathological museum, and, lastly, but by no manner of means least, the hospital wards. These are well worth inspection, not only because of the admirable way in which they are arranged, from the point of view of pure hospital construction only, but equally so because they represent an ideal in graduate hospital combinations, showing every possible department adequately provided for. In a general hospital this multiplicity of departments may be open to grave objection; in a graduate teaching institution such as this it is to be whole-heartedly praised, since it affords students every opportunity of becoming familiar with special work without being obliged to leave the precincts of the building. The orthopædic and children's wards are specially interesting, as they are new, and models of what such wards should be. Efficiently lighted, ventilated, and heated, they are in every way suitable for their intended purpose, and the economy of space, which is nowhere allowed to interfere with efficiency or with a proper regard for sound hygienic principles, that obtains in these wards is reminiscent of the Moabite Hospital at Berlin at its best. To some extent the hospital, as a building, must be regarded as not being new. Yet it is by no means old, and the few anomalies that, from the modern hospital designer's point of view, may be noticed are small and easily to be amended when the school decides to rebuild. The rebuilding scheme is under consideration, and active operations will be started as soon as the munificent legacy from the estate of the late

The New York Post-graduate Medical School and Hospital.

* The term "matriculate" as used in America is synonymous with our term "graduate," or "diplomate," meaning one who has actually got his degree or diploma.—Ed. The Hospital.

Mr. Hewitt, who bequeathed £400,000 ($2,000,000) to the institution, is available. At present the hospital expenditure is met by private subscription and by the fees derived from the school courses, which are generously presented to the main hospital fund by the professors and demonstrators. Some assistance is given by the City of New York, in the form of a small hospital grant, which is likely to be increased in the near future, when the institution takes a larger share in what may be called municipal hospital work than it is at present doing. American hospitals, especially in New York, are just now suffering from the depression consequent on the recent financial panic. The cost of supplies has increased, and the daily expenditure has risen in proportion. The Post-Graduate Hospital has also had to suffer, but it has pulled up manfully, and there is every reason to believe that, with the munificent Hewitt bequest to aid it, the extensive plan of development which the directors have in contemplation will be possible in the course of the present year.

During the past year the hospital treated, as in- and out-patients, a total of nearly 30,000 persons, of whom more than 75 per cent. were free patients. It is worth while to note that the institution makes a point of carefully investigating the claims of non-paying patients, by means of a system of inspection which has points in common with that in force at the Royal Free Hospital. No one is allowed to attend as a dispensary patient, or to occupy a "free" bed, who is able to pay even a nominal fee to an outside practitioner. Patients are received into the private rooms as paying patients and are charged according to their ability to pay. Taking the corporation statement for the past year, we find that the total expenditure was $236,078, while the total receipts fell short of that sum by $58,074.

A word must be said about the school for nurses, which is part of the hospital, though located in a separate block, fronting the Twentieth Street entrance to the main block. This is an excellently equipped building, with separate bedrooms, reception rooms, and a roof garden. The course of instruction is a thoroughly practical one, and the hospital offers exceptional advantages for the education and training of nurses. It is satisfactory to find that the post-graduate nurses are in great demand in the city and elsewhere, both for private and for institutional duty.

The hospital and school are managed by an influential corporation and by a faculty which is thoroughly representative. At the head of the executive is Dr. George N. Miller, while Dr. Arthur Chace acts as secretary. The superintendent of the hospital is Dr. Brush, to whose courtesy we are indebted for the opportunity of investigating the work and progress of the institution.

(*To be continued.*)

## Figures and Tables

**Figure f1:**